# Implementation of Electronic Decision Support for Diabetic Care in a Student-Run Clinic

**DOI:** 10.7759/cureus.12219

**Published:** 2020-12-22

**Authors:** Ankur Srivastava, Delia Shen, Maxim I Maron, Howard S Herman, Brandon S Cohen, Avigdor Nosrati, Amarilys R Cortijo, Sarah Nosal, Ellie Schoenbaum

**Affiliations:** 1 Internal Medicine, Albert Einstein College of Medicine, Bronx, USA; 2 Family Medicine, The Institute for Family Health, Bronx, USA

**Keywords:** diabetes, electronic medical record, clinical decision support

## Abstract

Background and objectives

Type 2 diabetes mellitus (T2DM) is a complex disease that can lead to complications. Electronic decision support in the electronic medical record (EMR) aids management. There is no study demonstrating the effectiveness of electronic decision support in assisting medical student providers in student-run free clinics.

Methods

There were 71 T2DM patients seen by medical students. Twenty-three encounters used a Diabetes Progress Note (DPN) that was created from consensus, opinion-based guidelines. Each note received a total composite score based on an eight-point scale for adherence to guidelines. Statistical comparisons between mean composite scores were performed using independent t-tests.

Results

The mean total composite score of DPN users was significantly greater than DPN non-users (5.35 vs. 4.23, p = 0.008), with a significant difference in the physical exam component (1.70 vs. 1.31, p = 0.002).

Conclusions

In this exploratory study, medical student providers at an attending-supervised, student-run free clinic that used electronic decision support during T2DM patient visits improved adherence to screening for diabetic complications and standard of care.

## Introduction

Located in the Bronx, New York, Einstein Community Health Outreach (ECHO) is an attending-supervised, student-run free clinic (SRC) affiliated with Albert Einstein College of Medicine (Einstein) and the Institute for Family Health (the Institute). ECHO is the first SRC in New York and has served as a model for other SRCs. ECHO provides health-care services for uninsured adults living in the Bronx and surrounding region, with approximately 1,000 patient visits annually. Third- and fourth-year medical students act as the primary patient provider with attending physicians supervising the patient assessment and plan. Third year students complete two clinic sessions during their family medicine clerkship while fourth year students volunteer for additional sessions with no differences in their role as medical student providers. For many patients, ECHO serves as an initial interface with the healthcare system. Annually, approximately 7% of these patients have Type 2 diabetes mellitus (T2DM), which is a chronic disease requiring longitudinal care in accordance with best clinical practices to prevent complications and progression of comorbidities [1].

Various organizations create consensus opinion-based guidelines for managing patients with diabetes, such as the American Diabetes Association (ADA), the American Association of Clinical Endocrinologists (AACE), American College of Endocrinology (ACE), and the National Kidney Foundation Kidney Disease Outcomes Quality Initiative (NKF KDOQI). These organizations recommend a series of referrals, physical examinations, and laboratory evaluations for routine patient visits if they have diabetes (summarized in the Appendix, Figure 3) [2-4].

However, previous studies have shown a lack of health care provider adherence to these guidelines in clinical practice due to time constraints and competing visit objectives when managing multiple patient issues [5]. It has been estimated that 45% of patients with diabetes fail to receive the recommended, evidence-based care [6]. Evidence has shown that provider-centric interventions, such as electronic decision support, could potentially improve provider adherence to standard of care and clinical outcomes by increasing screening rates for diabetic complications as well as improving blood pressure monitoring and glycemic control [7, 8].

The positive effects of electronic clinical decision support suggest that creating an outline for clinic visits will assist medical student providers in meeting standards of care and promoting clear documentation for future continuity of care. Here, we implement clinical decision support in the form of a Diabetes Progress Note (DPN) to facilitate medical student adherence to consensus opinion-based guidelines for patients with T2DM. Our exploratory study evaluates whether utilizing the DPN improves rates of medical student documentation and adherence to these screening and monitoring guidelines for patients with T2DM during the patients’ first ECHO visit.

## Materials and methods

**Figure 1:**
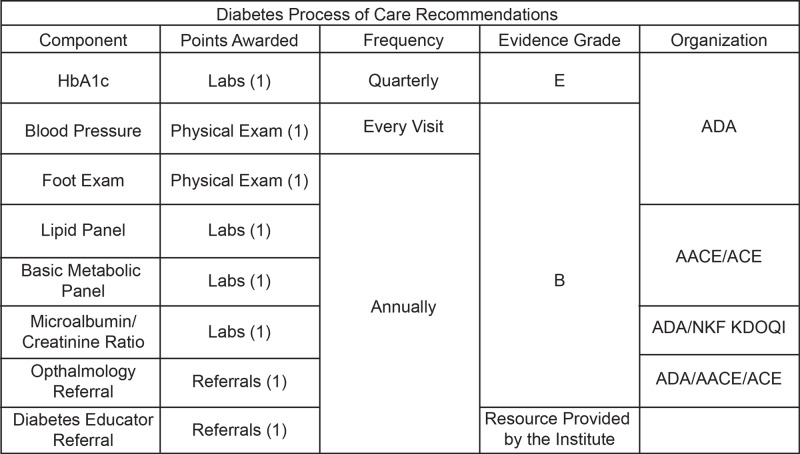
Consensus opinion-based guidelines on diabetes standard of care process variables with point allocation and further categorization. AACE: American Association of Clinical Endocrinologists; ACE: American College of Endocrinology; ADA: American Diabetes Association; NFK KDOQI: The National Kidney Foundation Kidney Disease Outcomes Quality Initiative.

Einstein and the Institute’s Institutional Review Boards approved this study (protocols 2014-3948 and 2242, respectively). A specialized DPN was incorporated into an existing progress note template, which helps medical students organize their patient visits at ECHO. The DPN was designed in accordance with consensus opinion-based guidelines for physical examination, laboratory evaluation, and referrals for ECHO patients with diabetes (Figure 1). The DPN had specific reminders for the frequency with which labs such as hemoglobin A1c, basic metabolic panel, lipid panel, and microalbumin to creatinine ratio had to be ordered. Similarly, reminders for checking the patient’s blood pressure at each visit and a yearly monofilament exam were placed in the physical exam portion of the DPN. Finally, there was a reminder in the DPN for a yearly ophthalmology referral and a referral to a diabetes educator, which is a service provided through the Institute to all patients with diabetes.

Starting in October 2017, third year medical students were trained on how to use the DPN during their orientation for their month-long family medicine clerkship (see the Appendix, Figure 4). Third- and fourth-year medical students were then given a brief refresher training at the beginning of every clinic day between October 2017 and October 2019. Medical students were instructed to use the DPN with patients who had an existing T2DM diagnosis gathered from patient history.

Each first encounter with a patient with T2DM was scored out of eight points, with one point given for successful documentation of each of the process quality variables (Figure 1). If there was no documentation of a process in the electronic medical record (EMR), it was assumed the process was not completed. The primary outcome was the composite score out of 8 (Figure 1). The first half of the academic year is defined as July to December and the second half of the academic year is defined as January to June.

**Figure 2:**
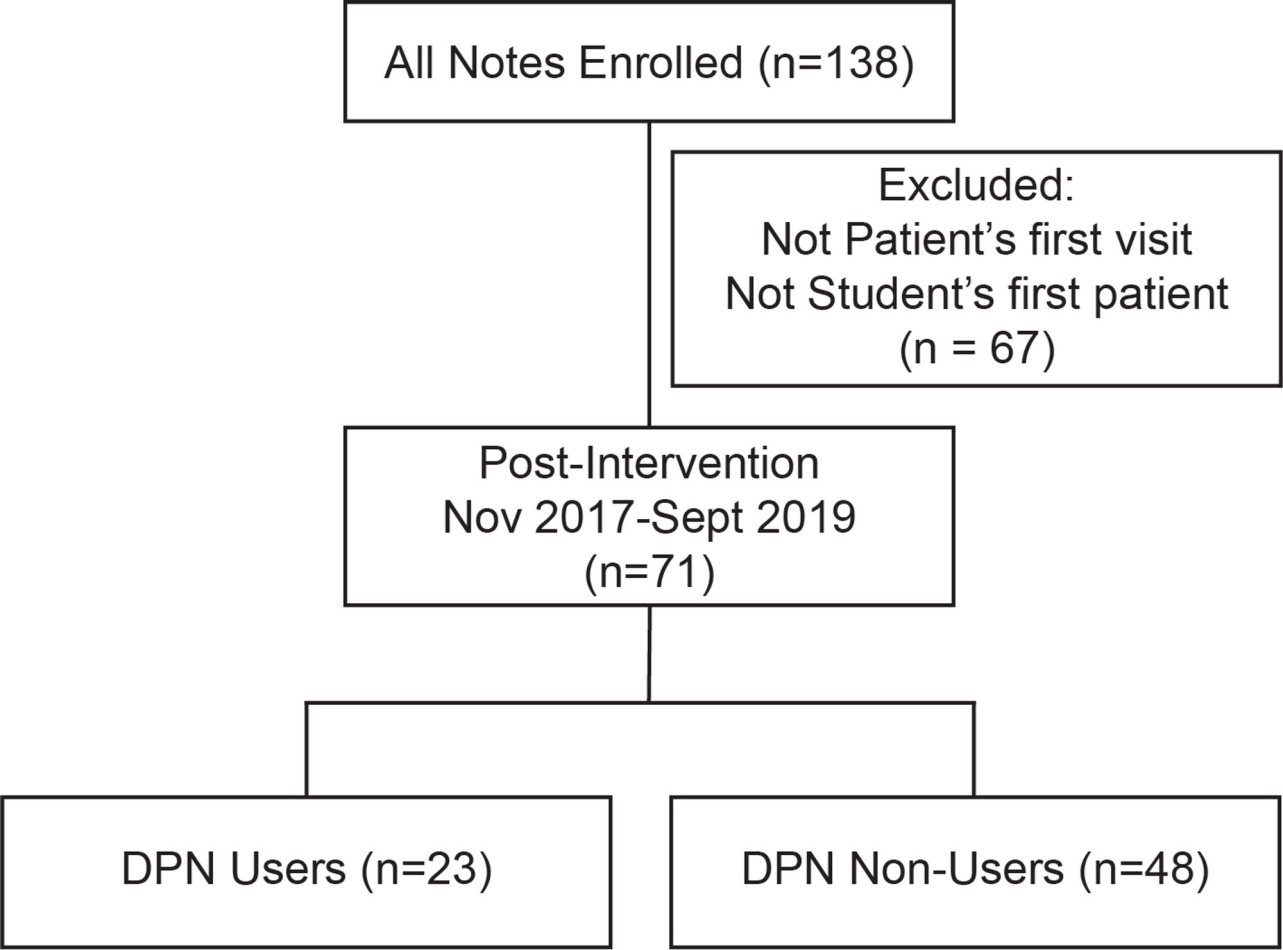
Flow chart diagramming the consolidation of medical students into study groups. DPN: Diabetes Progress Note

**Figure 3:**
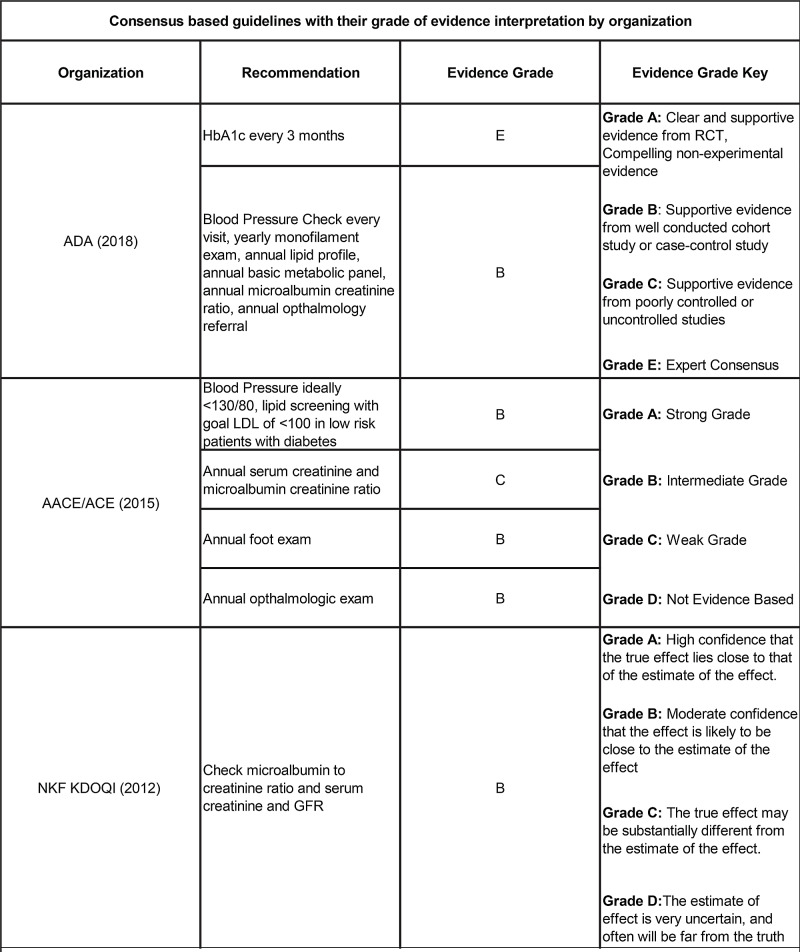
Consensus based guidelines with their grade of evidence interpretation by organization Consensus, opinion-based guideline recommendations with their respective evidence grade and interpretation. ADA: American Diabetes Association; AACE/ACE: American Association of Clinical Endocrinologists/American College of Endocrinology; NKF KDOQI: National Kidney Foundation Kidney Disease Outcomes Quality Initiative.

**Figure 4:**
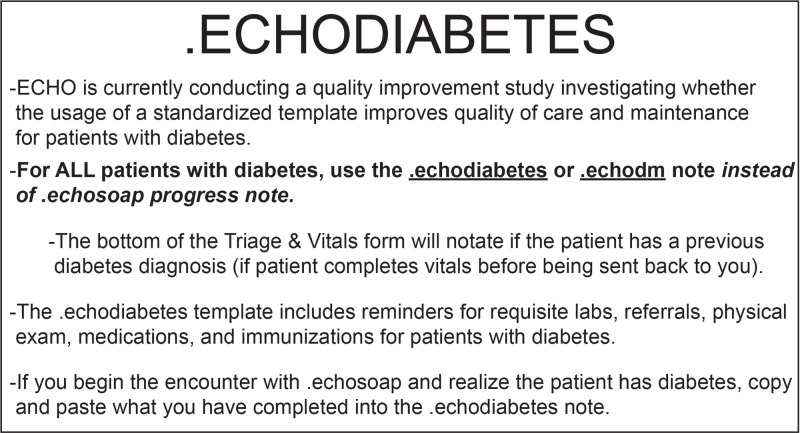
Instruction during medical student family medicine orientation for use of the Diabetes Progress Note.

All patients with Type 2 diabetes mellitus greater than the age of 18 were included in the study. If a patient with T2DM had more than one visit to ECHO, their first visit with the T2DM diagnosis was used and subsequent visit information was excluded in an effort to standardize the visit requirements (Figure 2). If the same medical student provider saw multiple patients, their first encounter was used, and their subsequent visit encounters were excluded to eliminate confounding experience with the DPN (Figure 2). Consequently, only patients that required all components of the DPN and who were seen by students with no prior DPN use were counted (Figure 2).

Data were collected from the EMR, de-identified and stored in REDCap, a Health Insurance Portability and Accountability Act (HIPAA) compliant database. Retrospective chart review was completed to identify unique patients with T2DM diagnosis and calculate their composite score on the eight-point scale. Those who used the DPN were designated DPN users and their peers, who did not use the DPN despite it being available to them, were designated as non-DPN users.

All statistical analysis was done using SPSS version 25.0 (IBM Corp., Armonk, NY). Patient and medical student provider demographic information was compared using Fisher’s Exact Test for discrete variables and independent t-tests for continuous variables. Results as composite scores were compared between study groups with independent t-tests.

## Results

Seventy-one patients with diabetes were seen at ECHO during the study period who met the study criteria. The DPN was used 23 times and was not used 48 times (Figure 2). Forty-two percent of patients in non-DPN user clinic visits were female as compared to 70% of patients in DPN user visits, which was significantly different (p = 0.042) (Table 1). There was no difference in the average age between the two groups (p = 0.123) (Table 1). While Hispanic patients made up the largest proportion of patients in both groups, there was no statistically significant difference in the racial/ethnic composition of the two groups (p = 0.318) (Table 1). Fifty-two percent of patients spoke Spanish in clinical encounters in non-DPN user visits versus 39% in DPN user visits (Table 1). Overall, there was no significant difference in language preference between the two groups (p = 0.339) (Table 1). When comparing the two groups’ past medical history, there were no significant differences in the rates of hypertension, hyperlipidemia, obesity or asthma (p = 0.613, 0.098, 0.287, 0.546, respectively) (Table 1).

**Table 1:** Patient demographic and past medical history information by DPN use. DPN: Diabetes Progress Note; PMH: Past Medical History; HTN: Hypertension; HLD: Hyperlipidemia; BMI: Body Mass Index.

	Patients with non-DPN User		Patients with DPN User		P-value
	n = 48		n = 23		
Female	20 (42%)		16 (70%)		0.042
Age	56.7 +/- 12.9		51.9 +/- 10.7		0.123
Race					0.3
Black	11 (23%)		8 (35%)		
Hispanic	29 (60%)		11 (48%)		
Other	4 (8.3%)		4 (17%)		
Language					0.3
English	22 (46%)		12 (52%)		
Spanish	25 (52%)		9 (39%)		
Other	1 (2%)		2 (9%)		
PMH					
HTN	27 (56%)		11 (48%)		0.613
HLD	11 (23%)		10 (43%)		0.098
Obesity	14 (29%)		10 (43%)		0.287
Asthma	1 (2.1%)		1 (4.3%)		0.546
BMI	29.08 +/- 4.874		31.19 +/- 10.304		0.263

The medical student provider composition was not significantly different (p = 0.078) (Table 2). Fifty-one percent of DPN non-users were third year medical students compared to 74% of DPN users (Table 2). The DPN was used significantly more in the second half of the academic year from January to June (p = 0.007) (Table 2).

**Table 2:** Medical student demographic information by DPN use status and DPN use timing across the year. MS: Medical Student; DPN: Diabetes Progress Note.

	Non-DPN User		DPN User		P-value
	n = 48		n = 23		
MS Year					0.078
Third	24 (51%)		17 (74%)		
Fourth	23 (47%)		6 (26%)		
Timing					0.007
Jan-Jun	32 (67%)		22 (96%)		
Jul-Dec	16 (33%)		1 (4%)		

Overall, the mean total composite score for DPN non-users was 4.23 with a median of 4 while the mean total composite score for DPN users was 5.35 with a median of 5, which was significantly different (p = 0.008) (Table 3). Similarly, when stratifying the data further, there was a significant difference (p = 0.002) in the mean physical exam composite score for DPN non-users (1.31) compared to DPN users (1.70) (Table 3). There was no significant difference between the labs composite score and referrals composite score when comparing DPN non-users to DPN users (p = 0.160, 0.068, respectively), although the referrals composite score trended towards significance (Table 3).

**Table 3:** Student performance by DPN use status. Student provider completion of consensus opinion-based process variables out of a total score of 8. Refer to Figure 1 for composite process variable point allocation. DPN: Diabetes Progress Note.

	DPN Non-User			DPN User			P-Value
	Mean	SD	Median	Mean	SD	Median	
Total	4.23	1.74	4	5.35	1.34	5	0.008
Labs	2.29	1.15	2.5	2.70	1.06	3	0.160
Physical Exam	1.31	0.47	1	1.70	0.47	2	0.002
Referrals	0.63	0.73	0	0.96	0.64	1	0.068

To assess confounding factors, composite scores by medical student provider year and time of year were compiled (Table 4). Medical student provider training did not have any effect on the total, labs, physical exam or referrals composite score (p = 0.800, 0.645, 0.375, 0.625, respectively) (Table 4). The total composite score in the second half of the academic year (4.75) was greater than the total composite score in the first half of the academic year (3.88), nearing statistical significance (p = 0.073) (Table 4). In the second half of the academic year, the mean labs composite score was 2.58, which is significantly different from the labs composite score in the first half of the academic year, 1.76 (p = 0.010) (Table 4). There was no significant difference in physical exam score or referrals score across time (p = 0.965, p = 0.843, respectively) (Table 4).

**Table 4:** Performance by medical student training or timing information. Student provider performance by confounding factors, such as medical student year or timing of Diabetes Progress Note use. Refer to Figure 1 for composite process variable point allocation. SD: Standard deviation.

	Third Year			Fourth Year			P-Value
	Mean	SD	Median	Mean	SD	Median	
Total	4.59	1.76	5	4.69	1.58	4	0.800
Labs	2.39	1.16	3	2.52	1.09	3	0.645
Physical Exam	1.49	0.51	1	1.38	0.49	1	0.375
Referrals	0.71	0.68	1	0.79	0.77	1	0.625

	January-June			July-December			P-Value
	Mean	SD	Median	Mean	SD	Median	
Total	4.75	1.71	5	3.88	1.69	4	0.073
Labs	2.58	1.07	3	1.76	1.25	2	0.010
Physical Exam	1.42	0.53	1	1.41	0.51	1	0.965
Referrals	0.75	0.7	1	0.71	0.772	1	0.843

## Discussion

Our study examined the effectiveness of using clinical decision support (the DPN) to assist medical student providers in adhering to standard of care for patients with diabetes. Overall, baseline patient characteristics were similar between patients in the DPN user and DPN non-user groups, with the majority of patients as Black or Hispanic with multiple chronic conditions including hypertension, hyperlipidemia and obesity (Table 1). This result suggests that DPN use was not biased by patient demographics, particularly because the prevalence of T2DM is higher in older patients as well as those that are Black or Hispanic as compared to non-Hispanic white [9]. Furthermore, the results revealed that medical students who utilized the DPN intervention were more likely to have increased adherence to T2DM standard of care process components, specifically in completing the physical exam components - including blood pressure and monofilament foot exam - when compared to their peers (Table 3).

Medical students transition from Einstein to work at ECHO from the end of May to early June of the following year. Those who have the family medicine rotation in the beginning half of the academic year from July to December may be more inexperienced in using the EMR and synthesizing clinical information into assessments and plans. Furthermore, fourth year medical students may be more experienced and more likely to initiate the DPN and follow through with the components listed compared to third year medical students. While there was more DPN utilization in the second half of the academic year (Table 2), this study’s results were independent of student training year or timing of year when looking at total composite score (Table 4).

The strength of our study lies in the novelty of assessing standardized documentation in the form of clinical decision support as a method to increase adherence of medical student providers to T2DM standard of care. It supports findings from other studies assessing the use of clinical decision support tools in improving clinical practice [10, 11].

The study also had limitations. This study did not randomize access to the DPN. Furthermore, our sample size was limited to those who used the DPN. Use of the DPN was dependent upon students initiating it within the EMR, despite reminders on the morning of clinic day resulting in a lower utilization rate. Future studies should automate the activation of the DPN in T2DM patient charts. There may also be an element of self-selection in the students that initiated use of the DPN when compared to those who did not. However, we lack baseline characteristics that could aid in understanding this phenomenon, such as level of prior training with the EMR. Future work will benefit from incorporating additional baseline characteristics as well as the perspective of students that did or did not use the DPN.

The EMR has changed how providers care for their patients. Many EMR systems have alerts incorporated into them to aid in clinical decision making, particularly for complex patients [12]. However, physicians routinely report that these reminders can be repetitive, confusing, or irrelevant, which suggests that the EMR can be perceived as a hindrance in patient care [13]. There is evidence to suggest that physicians stop responding to electronic decision support the longer they are exposed to it [14]. Medical student providers at ECHO have to sift through the EMR with their novice training. Electronic decision support may overwhelm medical students rather than support them, which is one possible reason why student providers did not opt to activate the DPN. However, students who did use the DPN performed significantly better than their peers in providing guideline-based care (Table 3). This suggests, that when done appropriately, embedding reminders in the medical note rather than a separate alert may be more effective in the EMR.

In the context of an attending-supervised, student-run free clinic, the DPN also serves as an educational tool. Literature on medical education has begun to explore the utility of the EMR as a part of student education [15]. This study further supports ways in which the EMR can be used to enhance medical education, particularly for students who are just beginning their clinical learning and transiently work in different clinical settings. Future studies should evaluate medical student knowledge before and after utilizing standardized documentation of guideline-based care as used in our intervention. As third- and fourth-year medical students begin to lay the foundation of their clinical knowledge and build illness scripts, the EMR can be used to support their training and enhance patient care. Furthermore, patients who visit an SRC are typically uninsured and have difficulty accessing continuous primary care. The DPN can serve as a tool to ensure all components of care are provided and improve documentation, thus enhancing continuity and coordination of treatment for our clinic’s population.

## Conclusions

This study represents the first, exploratory report of medical students at an attending-supervised, student-run free clinic successfully using standardized electronic decision support to improve adherence to screening for diabetic complications and evaluating patients with diabetes.
